# A human-centered automated machine learning agent with large language models for multimodal data management and analysis

**DOI:** 10.3389/frai.2025.1680845

**Published:** 2025-10-08

**Authors:** Rong Huang, Su Tao

**Affiliations:** ^1^Tawa Supermarket, Inc., Buena Park, CA, United States; ^2^University of California, Los Angeles, Los Angeles, CA, United States

**Keywords:** LLM, agent, AutoML, multimodal data analysis, deep learning

## Abstract

Automated Machine Learning (AutoML) aims to streamline the end-to-end process of ML models, yet current approaches remain constrained by rigid rule-based frameworks and structured input requirements that create barriers for non-expert users. Despite advances in Large Language Models (LLMs) demonstrating capabilities in code generation and natural language understanding, their potential to improve AutoML accessibility has not been fully realized. We present an innovative LLM-driven AI agent that enables natural language interaction throughout the entire ML workflow while maintaining high performance standards, reducing the need for predefined rules and minimizing technical expertise requirements. The proposed agent implements an end-to-end ML pipeline, incorporating automatic data loading and pre-processing, task identification, neural architecture selection, hyperparameter optimization, and training automation. Additionally, we propose a novel data processing approach that leverages LLMs to automatically interpret and handle diverse data formats without requiring manual pre-processing or format conversion. Moreover, we propose an adaptive hyperparameter optimization strategy that combines LLMs' knowledge of ML best practices with dynamic performance feedback to intelligently adjust search spaces. Extensive evaluation on 10 diverse datasets spanning classification and regression tasks across multiple data modalities demonstrates that our approach consistently achieves superior performance compared to traditional rule-based AutoML frameworks. By bridging the gap between human intent and ML implementation, our approach contributes to the development of a more accessible AutoML framework.

## 1 Introduction

Automated Machine Learning (AutoML) has emerged as a transformative approach to democratizing machine learning by automating the complex, time-intensive processes involved in model development and deployment (He et al., 2021; Barbudo et al., 2023; Lin et al., 2025). Although traditional ML workflows require extensive expertise in data pre-processing, architecture selection, and hyperparameter tuning, AutoML frameworks aim to make these capabilities accessible to users throughout the technical spectrum, from domain experts to experienced data scientists (Baratchi et al., 2024; Yao et al., 2018). The evolution of AutoML has seen advances in automating all components of the machine learning pipeline (Elshawi et al., 2019; Liu et al., 2024; Luo et al., 2024). These include automatic data pre-processing, model selection, training optimization, and hyperparameter tuning, all working in concert to reduce manual intervention while maintaining high performance standards (Khurana et al., 2016a). Current AutoML frameworks achieve the automation by different methods, from traditional heuristic approaches to advanced methods leveraging deep learning and evolutionary algorithms (Bahrami et al., 2022; Khurana et al., 2016b; Yang et al., 2020; Khurana et al., 2018), each offering distinct trade-offs between computational efficiency and model performance. Neural Architecture Search (NAS) represents one direction in the AutoML landscape, focusing on the algorithmic design of optimal neural network architectures for specific tasks (White et al., 2023). Recent innovations in NAS have improved search efficiency through the integration of reinforcement learning, evolutionary strategies, and gradient-based optimization methods (Liang et al., 2019; Elsken et al., 2017; Li and Malik, 2016; Pham et al., 2018; Zoph et al., 2018; Jin et al., 2019; Luo et al., 2018). Complementing these architectural advances, hyperparameter optimization (HPO) has evolved from simple grid and random search strategies to more sophisticated approaches using Bayesian optimization (Feurer and Hutter, 2019; Cakmak et al., 2020; Bae and Grosse, 2020), demonstrating superior performance in identifying optimal model configurations while minimizing computational overhead (Bergstra and Bengio, 2012; Yogatama and Mann, 2014).

Despite these advances in AutoML, current solutions remain constrained by rigid, rule-based frameworks that create substantial barriers for non-technical users. Moreover, underlying architectures of these AutoML methods reveal fundamental limitations in flexibility and accessibility. H2O.ai automates model selection and hyperparameter optimization (Candel et al., 2016), but its effectiveness depends heavily on predefined rules for data pre-processing and strict formatting requirements. Users must still possess technical knowledge to properly structure their data and configure the platform's parameters according to these predetermined rules. Auto-sklearn, though leveraging Bayesian optimization for scikit-learn models (Feurer et al., 2020), and AutoGluon, with its automated stack ensembling for multimodal data (Erickson et al., 2020), both operate within confined parameter spaces defined by fixed optimization strategies and predetermined model architectures. Similarly, Google's AutoML suite, despite offering specialized solutions for vision and natural language processing tasks (Bisong, 2019), and Hyperband's innovative multi-armed bandit strategy for hyperparameter optimization (Li et al., 2018), remain bound by rigid input specifications and predefined search spaces. These AutoML methods require users to conform to specific data formats, model configurations, and optimization procedures that cannot be easily modified or adapted to novel scenarios. Due to the implementations based on predefined rules, these constraints of existing AutoML methods manifest in several ways: (1) fixed dataset formats that limit data flexibility and usability for interaction, (2) predetermined model architectures that may not optimally suit unique problem spaces, and (3) rigid optimization strategies and hyper-parameter search space that cannot dynamically adapt to varying computational resources or performance requirements. Large Language Models (LLMs) have demonstrated capabilities in code generation and natural language understanding (Tornede et al., 2023), presenting a potential solution to these limitations through their ability to flexibly interpret user requirements and generate customized solutions. LLMs can transform AutoML through their ability to understand natural language descriptions of ML tasks, generate appropriate pre-processing code for diverse data formats, and dynamically adapt model architectures based on task requirements (Shen et al., 2024; Wang and Shen, 2024; Chen Z. et al., 2024). Their contextual understanding enables them to suggest suitable hyperparameters based on similar historical problems and adjust optimization strategies according to available computational resources. Furthermore, LLMs can provide natural language explanations of their decisions, making the AutoML process more transparent and interpretable (Zhang et al., 2024). However, their application in advancing AutoML remains largely unexplored.

These limitations collectively create three critical barriers that prevent AutoML from achieving its democratization goals: the fundamental incompatibility between rigid architectures and diverse real-world problems, the absence of adaptive optimization strategies that can respond to varying computational constraints and problem domains, and the lack of natural language interfaces accessible to domain experts without programming expertise. This research addresses these barriers by introducing an LLM-driven AutoML framework that leverages natural language understanding to eliminate rigid preprocessing requirements, implements adaptive optimization strategies informed by contextual task analysis, and enables intuitive human-machine interaction throughout the entire machine learning workflow.

This research addresses these limitations by introducing an innovative LLM-based agent that fundamentally reimagines the AutoML paradigm. Our approach harnesses the natural language understanding capabilities of LLMs to create a flexible, intuitive AutoML framework that “reduces reliance on predefined rules and largely abstracts away complex technical requirements.”

The major contributions of this work are three-fold. Firstly, we introduce an LLM-based agent that implements a complete AutoML pipeline through five integrated stages: automatic data loading and pre-processing, automatic task inference, dynamic model selection and construction, adaptive hyperparameter optimization, and automated training and evaluation. This end-to-end framework transforms how users interact with AutoML tools, enabling natural language communication throughout the entire ML workflow. Secondly, we present a novel data processing approach that leverages LLMs' contextual understanding to automatically interpret and handle diverse data formats. Unlike traditional AutoML methods that require strict data formatting and schema definitions, our method can dynamically analyze raw data structures, infer relationships between variables, and automatically generate appropriate pre-processing pipelines. This innovation enables the AutoML framework to work with unstructured text files, semi-structured JSONs, various tabular formats, and even mixed data types without requiring manual pre-processing or format conversion. Finally, we introduce an adaptive hyperparameter optimization strategy that combines LLM's capacity to generate contextually appropriate machine learning code and recommendations based on patterns learned from extensive training data including machine learning literature, documentation, and code repositories with dynamic performance feedback. Traditional AutoML approaches rely on fixed optimization strategies and predefined parameter spaces. In contrast, our approach leverages LLMs to analyze the specific characteristics of each task, suggest initial hyperparameter configurations based on similar historical problems, and dynamically adjust the search space based on intermediate training results.

This manuscript is structured as follows. Section 2 presents a literature review that examines existing AutoML frameworks, multimodal data processing systems, and applications of LLMs in automated programming contexts, establishing the theoretical foundation and identifying research gaps that motivate our approach. Section 3 details our methodology through five subsections that describe the overall framework architecture, data preprocessing and task inference mechanisms, model selection strategies, adaptive hyperparameter optimization approaches, and automated code generation processes. Section 4 provides extensive experimental evaluation beginning with dataset descriptions and evaluation metrics, followed by implementation details, comprehensive results analysis including performance comparisons with baseline methods, computational efficiency assessments, scalability studies across varying dataset sizes, hyperparameter optimization convergence analysis, resource utilization evaluations, and ablation studies that isolate the contribution of individual framework components. Section 5 addresses important limitations of our approach and discusses ethical considerations related to bias propagation, privacy concerns, and responsible deployment practices. Section 6 concludes the manuscript by summarizing key contributions, discussing implications for the broader AutoML research community, and outlining promising directions for future research including multimodal extensions and interpretability enhancements.

## 2 Related work

### 2.1 Automated machine learning frameworks

The field of AutoML has evolved over the past decades. Early commercial solutions in the 1990s offered automatic hyperparameter optimization for classification algorithms via grid search (Dinsmore, 2016). The formalization of the combined algorithm selection and hyperparameter optimization (CASH) problem by Thornton et al. (2013) marked a pivotal moment in AutoML research. Modern AutoML frameworks can be broadly categorized into two paradigms: those employing fixed pipeline structures and those supporting variable structures. Fixed-structure frameworks, such as Auto-sklearn (Feurer et al., 2015), TPOT (Olson et al., 2016), and H2O AutoML (LeDell and Poirier, 2020), implement predefined sequences of data cleaning, feature selection, preprocessing, and modeling steps. While these frameworks reduce complexity by eliminating structure search, they may yield suboptimal performance for complex datasets requiring specialized preprocessing pipelines (Zöller and Huber, 2021).

Variable-structure approaches offer greater flexibility through dynamic pipeline construction. Genetic programming-based methods (Olson et al., 2016) interpret pipelines as tree structures that evolve through crossover and mutation operations. Hierarchical task networks (Mohr et al., 2018) decompose complex pipeline construction into manageable subproblems, while Monte Carlo tree search approaches (Rakotoarison et al., 2019) iteratively build pipelines of increasing complexity. Reinforcement learning methods, such as the self-play approach by Drori et al. (2018), model pipeline construction as a game where agents learn optimal strategies through iterative improvement. We provide a high-level comparison of existing AutoML framework in Table 1.

**Table 1:** Comparison of AutoML frameworks.

**Framework**	**Flexibility**	**User interface**	**Data support**	**Performance**	**Strengths**	**Limitations**
Auto-sklearn	Medium	Programmatic API	Tabular only	High	Bayesian optimizationMeta-learning Ensemble methods	Fixed pipeline structureLimited data format support Requires ML knowledge
AutoGluon	High	Simple API	Tabular, Text, Images, Multimodal	Very high	Stack ensembling Multimodal support Strong performance	High computational costLimited interpretability Complex deployment
H2O AutoML	Medium	GUI + API	Tabular, Time series	High	User-friendly interface Enterprise features Good documentation	Rigid preprocessing rules Format constraints Limited customization
TPOT	Very high	programmatic API	tabular only	medium	Pipeline evolution High flexibility Open source	Long optimization time Complexity for beginners Genetic programming overhead
AutoKeras	medium	simple API	images, text, structured data	medium	Easy to use Neural architecture search TensorFlow integration	Limited architecture diversity TensorFlow dependency Moderate performance
Google AutoML	low	web interface	images, text, tables, video	high	No coding required Cloud integration Professional support	Expensive Limited customization Vendor lock-in
MLBox	medium	programmatic API	tabular, time series	medium	Automated preprocessing Feature selection Cross-validation	Limited algorithm support Basic optimization Documentation gaps
**Our Framework**	**Very high**	**natural language**	**all formats multimodal unstructured**	**very high**	**Natural language interface Intelligent automation Adaptive optimization**	**LLM dependency Generation variability External API requirements**

Performance ratings indicate typical predictive accuracy and model quality achieved across diverse machine learning tasks based on published benchmark studies.

### 2.2 Multimodal AutoML systems

The growing importance of multimodal data has spurred development of specialized AutoML frameworks. AutoGluon-Multimodal (Erickson et al., 2020) extends traditional tabular AutoML to handle text, images, and mixed-modal datasets through automated preprocessing and model selection. Recent work on multimodal pipeline synthesis (Shi et al., 2024) leverages pre-trained transformer models to unify diverse data modalities into high-dimensional embeddings.

Vision-language pre-trained models have emerged as powerful foundations for multimodal AutoML (Radford et al., 2021). These models enable zero-shot classification and few-shot adaptation across diverse visual and textual domains, reducing the need for extensive task-specific training data.

### 2.3 Natural language processing applications in AutoML

AutoML applications in natural language processing face unique challenges due to the complexity and variability of textual data. Automated text preprocessing, including tokenization, normalization, and encoding selection, requires an understanding of linguistic structures (Wolf et al., 2020; Han et al., 2025). Recent work on automated text summarization (Zhang et al., 2020), natural language inference (Conneau et al., 2017), and open-domain question answering (Karpukhin et al., 2020) demonstrates the potential for AutoML in complex NLP tasks. Zero-shot and few-shot learning approaches (Brown et al., 2020) enable rapid adaptation to new domains without extensive retraining. The emergence of instruction-tuned models (Wei et al., 2022a) and chain-of-thought reasoning (Wei et al., 2022b; Shen et al., 2025a,b,c) provides new opportunities for incorporating natural language understanding directly into AutoML workflows, enabling more intuitive human-AI interaction during the machine learning development process.

However, these existing applications of natural language processing in AutoML contexts represent a fundamentally different paradigm from our proposed approach. Traditional NLP-enhanced AutoML systems typically employ natural language processing as a specialized component within otherwise rule-based frameworks, focusing on specific tasks such as text classification, summarization, or question answering. In contrast, our framework positions the LLM as the central decision-making entity that orchestrates and governs the entire AutoML pipeline through continuous natural language reasoning and code generation. The key distinction lies in the scope and nature of LLM integration. Existing approaches in this domain can be categorized into three primary patterns: task-specific NLP automation, where natural language processing techniques are applied to automate particular aspects of text-based machine learning tasks; hybrid NLP-AutoML systems, where traditional AutoML frameworks are augmented with NLP capabilities for handling textual data; and instruction-following systems, where pre-trained language models are fine-tuned to follow specific commands within constrained AutoML workflows. Our approach diverges from these patterns by implementing what we term “LLM-native AutoML,” where the language model serves as the primary reasoning engine for all pipeline decisions, from data preprocessing and task inference to model selection and hyperparameter optimization. Rather than using LLMs to enhance specific components of traditional AutoML frameworks, our system relies on the LLM's contextual understanding and code generation capabilities to dynamically construct and adapt the entire machine learning workflow. This paradigm shift enables our framework to handle previously unsupported scenarios such as unstructured data interpretation, cross-modal reasoning, and adaptive optimization strategies that evolve based on intermediate results.

### 2.4 LLM-based AutoML frameworks

Recent LLM-enhanced AutoML frameworks adopt different architectures that our approach addresses through a unified design. Moharil et al. (2024) leveraged pre-trained transformers as feature extractors for multimodal pipeline synthesis within traditional Bayesian optimization frameworks, but operate within predefined architectural constraints that limit adaptability to novel data types. Chen S. et al. (2024) proposed template-bounded methods that ensure code executability by constraining LLM outputs within predetermined structures, prioritizing reliability over the flexibility needed for diverse problem scenarios. Trirat et al. (2024) introduced a multi-agent architecture in which specialized LLM agents collaborate through retrieval-augmented planning, enabling parallelization but introducing coordination complexity and potential inconsistencies between interdependent pipeline decisions.

## 3 Methods

### 3.1 Method overview

**Figure 1:**
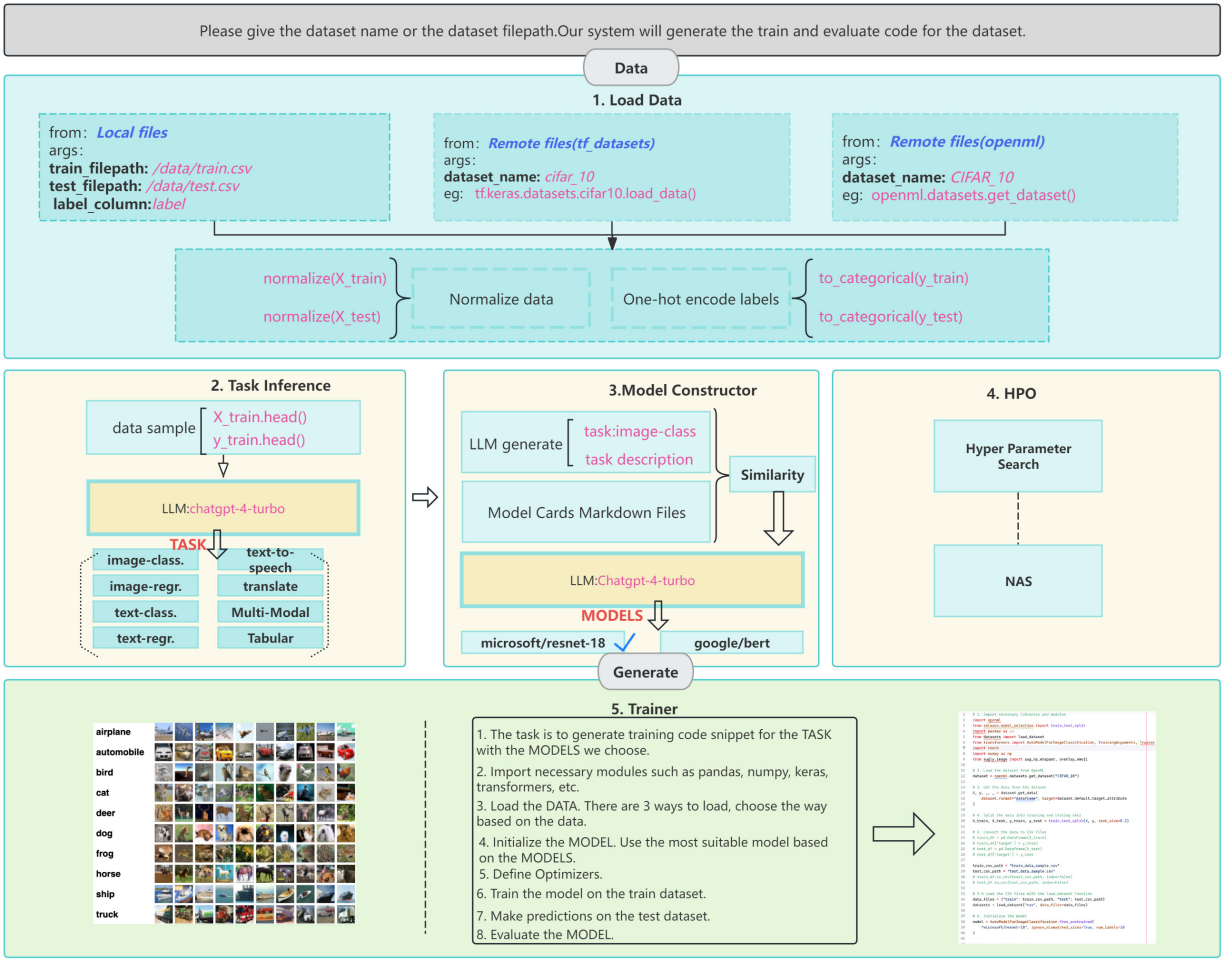
Illustration of the proposed AutoML pipeline. The framework encompasses five main stages: (1) Data loading and pre-processing, supporting both local and remote data sources (deep blue text); (2) Task inference using LLMs to analyze data characteristics (light blue boxes); (3) Model selection, leveraging LLMs and model cards to identify suitable architectures (yellow box with blue border); (4) HPO, including both hyperparameter search and NAS; and (5) Automated training and evaluation code generation. Pink text exemplifies specific implementations, while bold black text under “args” indicates input parameters. Model names are shown in deep red.

Our proposed LLM-driven AutoML framework implements an end-to-end pipeline that transforms traditional rule-based automation into a flexible, natural language-guided process. The framework operates through five integrated stages that work in concert to deliver a comprehensive ML solution while maintaining accessibility for non-expert users, as shown in Figure 1. The pipeline begins with automatic data loading and pre-processing, where the LLM leverages its contextual understanding to interpret diverse data formats and structures with minimal manual intervention and reduced reliance on predetermined schema. This stage dynamically generates appropriate pre-processing pipelines based on the input data's characteristics, handling everything from unstructured text to mixed-type tabular data. Following data preparation, the task inference stage employs the LLM to analyze the dataset and problem context, determining the appropriate ML paradigm (e.g., classification, regression, clustering and etc.) and any specific requirements or constraints. This analysis forms the foundation for subsequent architectural decisions and optimization strategies. The model construction stage then utilizes the LLM's extensive knowledge base in conjunction with model card specifications to select and configure appropriate architectures. This process considers multiple factors including the identified task requirements, dataset characteristics, and available computational resources to ensure optimal model selection. The fourth stage implements our adaptive hyperparameter optimization strategy, which uniquely combines the LLM's understanding of ML best practices with dynamic performance feedback. This approach moves beyond traditional fixed search spaces by suggesting initial configurations based on similar historical problems and continuously adjusting the optimization strategy based on intermediate training results. Finally, the training and evaluation stage automatically generates and executes appropriate code for model training, validation, and testing.

To illustrate the framework's operation, consider a user providing a CSV file containing customer transaction data with the instruction “*predict customer churn*.” The workflow proceeds as follows: First, in the data preprocessing stage, the LLM examines the data structure and generates analysis such as “*The dataset contains 15 features including categorical variables (customer_type, payment_method) with high cardinality requiring label encoding, numerical features (transaction_amount, account_balance) showing right-skewed distributions necessitating log transformation, and temporal features (last_transaction_date) requiring datetime parsing and feature extraction*.” The LLM then generates corresponding preprocessing code implementing these transformations. Second, during task inference, the LLM analyzes the target variable and data characteristics, concluding “*Based on the binary target variable (churned: 0/1) and feature distributions, this is a binary classification task with moderate class imbalance (70%/30% ratio), suggesting the need for stratified sampling and weighted loss functions*.” Third, in model selection, the LLM reasons “*Given the tabular nature, moderate dataset size (10K samples), and mixed feature types, gradient boosting models like XGBoost or ensemble methods would be most suitable, as they handle feature interactions well and are robust to different data types*.” Fourth, for hyperparameter optimization, the LLM provides informed starting points: “*For XGBoost on this imbalanced classification task, I recommend starting with learning_rate* = *0.1, max_depth* = *6, subsample* = *0.8, and scale_pos_weight* = *2.33 to address class imbalance, then exploring ranges of [0.05–0.3] for learning rate and [3–10] for max depth*.” Finally, the LLM generates complete training and evaluation code incorporating these decisions, including appropriate metrics like F1-score and AUC-ROC for the imbalanced classification task.

### 3.2 Data pre-processing and task inference

The first stage of our framework improves traditional AutoML data handling by implementing an LLM-driven approach that automatically interprets and processes diverse data formats while inferring appropriate ML tasks. Specifically, we implement a data loading module that can handle both local and remote data sources through a natural language interface. For local files, it accepts various formats including CSV, JSON, and unstructured text files, requiring only minimal input, namely the training data location, testing data location, and a natural language description of the target variable.

#### 3.2.1 Data pre-processing

The pre-processing pipeline harnesses the LLM's contextual understanding through a multi-step analysis process. Initially, we prompt the LLM to examine a sample of the input data to identify data types, structural patterns, and potential relationships between variables, which generates a structured representation of the dataset's characteristics, including feature distributions, missing value patterns, and potential correlations. Afterwards, we prompt the LLM to translate this understanding into executable pre-processing code following a given template, which incorporates best practices for data cleaning and transformation. For example, in terms of numerical features, we first analyze distribution characteristics including skewness, kurtosis, and the presence of outliers. Based on these metrics, we select appropriate scaling methods, e.g., implementing min-max normalization for bounded distributions, standard scaling for approximately normal distributions, and robust scaling for outlier-heavy features. Similarly, for categorical variables, we employ a decision tree architecture that considers cardinality, semantic relationships, and memory constraints to choose between encoding strategies. Variables with low cardinality are typically one-hot encoded, while high-cardinality features may use label encoding or learned embeddings to maintain efficiency. Our data loading module also supports seamless integration with established data repositories, including TensorFlow datasets and OpenML, automatically handling format conversions and pre-processing requirements.

#### 3.2.2 Context-aware task inference

The task inference module leverages the LLM's understanding of ML paradigms to determine optimal modeling approaches. Specifically, it employs a three-stage analysis process to examine the pre-processed data and generate detailed task specifications. Firstly, it performs statistical analysis through a series of automated tests, including feature-wise distributions, variance analysis, and higher-order moments. It employs mutual information scoring to quantify relationships between features and target variables, while utilizing correlation matrices and chi-square tests to detect dependencies among variables. In the second stage, we prompt the LLM to processes these statistical insights to generate task specifications. For example, when examining class distributions in classification tasks, the LLM calculates class ratios and determines imbalance severity using predefined thresholds. The analysis generates structured output detailing the primary task type (e.g., binary classification, multi-class classification, and regression) along with specific subtasks and constraints. The final stage focuses on generating comprehensive modeling recommendations. The LLM maps data characteristics to specific modeling strategies. For instance, when detecting severe class imbalance (ratio >1:10), it automatically recommends techniques such as SMOTE for augmentation or weighted loss functions.

### 3.3 Model selection

The model selection stage combines a comprehensive model knowledge base with LLM-driven analysis to identify optimal architectures for given tasks. This stage leverages both the detailed task specifications from the inference stage and a curated pool of state-of-the-art models to make informed selection decisions.

#### 3.3.1 Dynamic model knowledge base

We construct a model knowledge base by aggregating and structuring model card information from Hugging Face's repository. Each model card is processed to extract metadata including architectural details, performance characteristics, resource requirements, and documented use cases. Our framework maintains this knowledge base through an automated updating mechanism that periodically incorporates new models and updates existing information. To ensure efficient retrieval, we implement a dense vector index of the model cards using a pre-trained encoder using BGE-M3.

#### 3.3.2 Model selection process

We implement a three-phase selection process that leverages both LLM capabilities and semantic similarity metrics to identify optimal model architectures. In the first phase, our framework generates dense vector representations of both the task specifications and model cards using BGE-M3 to enable efficient initial filtering through cosine similarity computations. The second phase employs a scoring mechanism where the LLM analyzes the top-k models identified through semantic similarity. The scoring considers multiple factors including computational requirements, model complexity, and historical performance on similar tasks. The LLM generates a structured analysis for each candidate model, evaluating its suitability across multiple dimensions such as input compatibility, output structure, and alignment with task-specific constraints. The final selection phase implements a ranking algorithm that combines both quantitative metrics and qualitative assessments. We weight various factors including semantic similarity scores, computational efficiency, and the LLM's detailed analysis uniformly to generate a final ranking. Consequently, we obtain a list of N candidate models, where N is configurable based on user requirements. For each selected model, we obtain a detailed justification of its selection correspondingly from the LLM, including specific strengths and potential limitations for the given task.

### 3.4 Hyperparameter optimization

Our framework implements an innovative hyperparameter optimization approach that combines traditional search strategies with LLM-driven intelligence to efficiently identify optimal model configurations.

#### 3.4.1 Adaptive search strategy

The optimization process begins with an initialization phase where the LLM analyzes the selected model architecture and task characteristics to generate informed starting points. Using its extensive knowledge of ML, the LLM identifies typical hyperparameter ranges and their interdependencies, creating a structured search space that reflects meaningful parameter combinations. This initial configuration serves as a foundation for subsequent optimization. Our agent then implements a hybrid search mechanism that integrates random exploration with Bayesian optimization. The random component employs Latin hypercube sampling to ensure uniform coverage of the search space while maintaining diversity in parameter combinations. Concurrently, the Bayesian optimizer utilizes a Gaussian Process model to construct a probabilistic surrogate of the objective function, enabling efficient identification of promising regions in the hyperparameter space.

#### 3.4.2 Resource-aware optimization

Our framework incorporates an adaptive resource allocation strategy inspired by the Hyperband algorithm. This approach dynamically adjusts the computational budget for each configuration evaluation based on performance trajectories. We implement a successive halving mechanism where poorly performing configurations are terminated early, allowing reallocation of resources to more promising candidates. To enhance efficiency further, we implement a meta-learning that maintains a database of previous optimization results across similar tasks. This historical data informs the construction of task-specific priors for the Bayesian optimizer and guides the selection of initial configurations.

#### 3.4.3 LLM-enhanced parameter tuning

The LLM performs continuous analysis of intermediate training results, generating structured insights about parameter sensitivity and interaction effects. This analysis drives adaptive adjustment of the search strategy, including modification of parameter ranges and exploration-exploitation trade-offs. Our framework implements a multi-objective optimization framework that considers multiple performance metrics simultaneously. The LLM aids in this process by analyzing task requirements and user preferences to construct appropriate objective functions. These functions incorporate various metrics including model accuracy, inference time, and resource utilization, enabling the discovery of Pareto-optimal solutions that balance competing objectives. Furthermore, the LLM implements an intelligent early stopping mechanism by analyzing training trajectories and comparing them with patterns observed in successful optimization runs.

### 3.5 Generation of training and evaluation code

Our framework utilizes carefully designed prompts that guide the LLM in generating appropriate code for each unique combination of data, task, and model. For instance, when dealing with a dataset like CIFAR-10, we first instruct the LLM to generate training and evaluation code specifically for the given task and model. Specifically, the LLM begins by importing essential libraries and modules, such as pandas, numpy, keras, sklearn, and task-specific libraries like transformers for NLP tasks. Next, the code for data loading is generated, accommodating the various data sources supported by our system (local files, TensorFlow datasets, or OpenML datasets). The LLM adapts this step based on the specific data loading method used in earlier stages. The model initialization follows, with the LLM generating code to instantiate the selected model architecture. The LLM then defines appropriate optimizers and loss functions, tailoring these choices to the specific task and model architecture. This includes generating code for custom loss functions if required by the task. For the training process, the LLM produces code that efficiently trains the model on the provided dataset. This includes setting up training loops, implementing batch processing, and incorporating any necessary callbacks or learning rate schedules. Finally, the LLM generates code for model evaluation, including making predictions on the test set and calculating relevant performance metrics.

## 4 Experiments

### 4.1 Datasets and evaluation metrics

We curated a diverse set of datasets to thoroughly assess our framework's performance across various tasks, data modalities, and complexities. The datasets were sourced from multiple repositories, including scikit-learn, OpenML, Hugging Face Datasets, TensorFlow Datasets. For binary classification tasks, we employed the following datasets. The Breast Cancer Wisconsin (Diagnostic) Dataset, sourced from scikit-learn, contains 569 instances with 30 features derived from digitized images of breast mass, aiming to classify tumors as malignant or benign. From OpenML, we utilized the Blood Transfusion Service Center Dataset (Yeh et al., 2009) with 748 instances and 5 features related to blood donation history, and the German Credit Dataset (Credit-g) comprising 1,000 instances and 20 features for credit risk assessment. The Phishing Websites Dataset (Mohammad et al., 2012), also from OpenML, includes 11,055 instances with 30 features to distinguish between legitimate and phishing websites. The Pima Indians Diabetes Dataset (Smith et al., 1988), another OpenML dataset, contains 768 instances with 8 medical features to predict diabetes likelihood. Lastly, we included the Titanic Dataset, containing information on 891 passengers with 12 features to predict survival. For multi-class classification, we employed two image datasets. The MNIST dataset, sourced from Hugging Face Datasets, consists of 70,000 28 × 28 grayscale images of handwritten digits (0–9). We also used the Fashion-MNIST dataset (Xiao et al., 2017), a local dataset comprising 70,000 28 × 28 grayscale images of fashion items across 10 categories, serving as a more challenging alternative to the original MNIST. To evaluate regression tasks, we utilized the Red Wine Quality and White Wine Quality datasets from OpenML. Both datasets contain physicochemical features of wine samples, with the Red Wine dataset including 1,599 instances and the White Wine dataset containing 4,898 instances. Each dataset has 11 features, and the task is to predict the wine quality score on a scale from 0 to 10. Classification tasks are assessed using accuracy, while regression tasks utilize Root Mean Square Error (RMSE).

### 4.2 Implementation details

Our framework is implemented using GPT-4-Turbo (gpt-4-turbo-2024-04-09) as the backbone LLM. We selected GPT-4-Turbo based on several key factors that align with AutoML requirements: superior code generation capabilities demonstrated across multiple programming languages and machine learning frameworks, extensive knowledge of current ML best practices and algorithms, robust reasoning abilities for complex multi-step decision making, and reliable performance in structured output generation required for our automated pipeline. GPT-4-Turbo's 128k context window enables processing of large datasets and comprehensive model documentation, while its training data cutoff includes recent developments in machine learning libraries and methodologies. We utilize the model with temperature = 0.1 to ensure consistent and deterministic outputs across runs, and implement structured prompting strategies that leverage the model's instruction-following capabilities for reliable code generation and analysis.

## 5 Results

Table 2 presents the comparative performance of our LLM-based AutoML framework against two rule-based AutoML methods: AutoGluon and AutoKeras. Our framework consistently outperforms both AutoGluon and AutoKeras across all datasets, demonstrating its versatility and effectiveness. In binary classification tasks, our method achieves superior performance. For the BreastCancer dataset, we attain an accuracy of 0.9649, surpassing AutoGluon (0.9590) and AutoKeras (0.9520). Similarly, for the Blood Transfusion dataset, our framework achieves an accuracy of 0.7600, outperforming both AutoGluon (0.7467) and AutoKeras (0.7500). The Credit-g dataset shows a similar trend, with our method achieving 0.7900 accuracy compared to AutoGluon's 0.7850 and AutoKeras's 0.7200. For multi-class image classification tasks, our framework demonstrates improvements. On the FashionMNIST dataset, we achieve an accuracy of 0.9250, notably higher than AutoGluon (0.9122) and AutoKeras (0.9111). The MNIST dataset shows similar results, with our method reaching 0.9940 accuracy, compared to AutoGluon's 0.9856 and AutoKeras's 0.9790. The Titanic dataset presents a particularly striking result, with our method achieving perfect accuracy (1.0000), substantially outperforming both AutoGluon (0.7765) and AutoKeras (0.7841). This perfect classification performance, while remarkable, is explainable given the dataset's characteristics and our framework's capabilities. The Titanic dataset represents a well-structured classification problem with 891 instances containing highly informative features such as passenger class, gender, age, and fare that exhibit strong predictive relationships with survival outcomes. Our LLM-driven AutoML approach achieves this optimal performance through several mechanisms: (1) intelligent feature engineering that creates composite variables capturing interactions (such as the combination of passenger class and gender), (2) preprocessing that optimally handles missing values and categorical encodings, and informed model selection that identifies architectures particularly suited to this type of tabular classification task. The LLM's extensive knowledge of machine learning enables it to recognize and implement domain-specific optimizations that traditional rule-based AutoML methods typically miss, such as creating age-group categorical features or applying class-specific transformations. This result demonstrates our AutoML framework's ability to achieve optimal performance when sufficient predictive signal exists in the data, highlighting the advantage of knowledge-driven automation over systematic search approaches. For regression tasks, our framework also shows superior performance. In the Diabetes dataset, our method achieves an RMSE of 0.3999, lower than AutoGluon (0.5222) and AutoKeras (1.8411). The wine quality prediction tasks (RedWine and WhiteWine) also show improvements, with our method achieving lower RMSE values compared to both baseline methods.

**Table 2:** Comparative performance of AutoML frameworks.

**Datasets**	**AutoGluon**	**AutoKeras**	**Ours**
BreastCancer (↑)	0.9590	0.9520	**0.9649**
FashionMNIST (↑)	0.9122	0.9111	**0.9250**
MNIST (↑)	0.9856	0.9790	**0.9940**
Titanic (↑)	0.7765	0.7841	**1.0000**
Blood (↑)	0.7467	0.7500	**0.7600**
Credit-g (↑)	0.7850	0.7200	**0.7900**
Phishing (↑)	0.9617	0.9466	**0.9647**
Diabetes (↓)	0.5222	1.8411	**0.3999**
RedWine (↓)	0.6225	1.0885	**0.6087**
WhiteWine (↓)	0.6349	0.7388	**0.6335**

Results show accuracy for classification tasks (↑) and RMSE for regression tasks (↓) across diverse datasets. Our LLM-based AutoML framework is compared against rule-based AutoML methods, i.e., AutoGluon and AutoKeras. The arrow symbols (↑, ↓) indicate whether higher or lower values are better for each metric. The bold values represent the best performance results across all compared frameworks for each dataset.

All the results show a statistic significance with *p* < 0.05. Statistical significance of performance differences was evaluated using paired t-tests comparing our framework against each baseline method across all datasets, with Bonferroni correction applied to control for multiple comparisons (α = 0.05). These results demonstrate that our LLM-based AutoML framework can effectively adapt to various tasks and data types, consistently outperforming rule-based AutoML methods. The performance improvements are observed across different data modalities (tabular and image data) and task types (binary classification, multi-class classification, and regression), highlighting the versatility and effectiveness of our approach in automating the end-to-end machine learning pipeline.

### 5.1 Computational efficiency analysis

**Figure 2:**
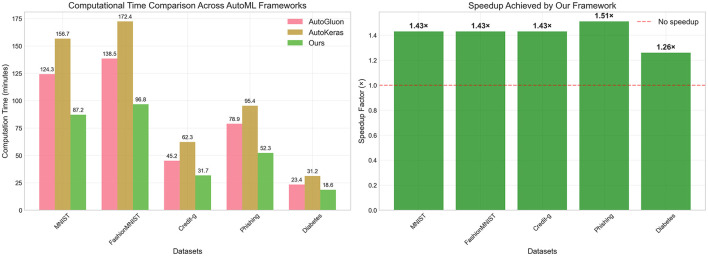
Computational efficiency analysis of AutoML frameworks. **(Left)** Shows absolute computation time (in minutes) required for complete AutoML pipeline execution across five representative datasets, comparing AutoGluon, AutoKeras, and our LLM-based framework. **(Right)** Displays the speedup factor achieved by our framework relative to the average performance of baseline methods, with speedup values ranging from 1.26× to 1.51× across different datasets. The horizontal dashed line at 1.0× indicates no speedup for reference.

Beyond performance improvements, computational efficiency represents an important factor in practical AutoML deployment. We conducted a timing analysis to evaluate the computational overhead of our LLM-based approach compared to traditional rule-based AutoML frameworks. Figure 2 presents the computational time comparison across five representative datasets, measuring the complete end-to-end pipeline execution from data loading to final model evaluation, we compare the speed up to AutoKeras. The results demonstrate that our LLM-based framework achieves substantial computational efficiency gains across all evaluated datasets. The absolute computation times, shown in the left panel of Figure 2, reveal consistent reductions compared to both AutoGluon and AutoKeras. For instance, on the MNIST dataset, our framework requires only 87.2 min compared to AutoGluon's 124.3 min and AutoKeras's 156.7 min. Similarly, for the Credit-g dataset, our method completes the pipeline in 31.7 min, substantially faster than AutoGluon (45.2 min) and AutoKeras (62.3 min). The speedup analysis, presented in the right panel of Figure 2, quantifies these efficiency improvements. Our framework achieves speedup factors ranging from 1.26× to 1.51× across different datasets, with the most significant acceleration observed on the Phishing dataset (1.51× speedup). The consistent speedup across diverse datasets indicates that the efficiency gains are not dataset-specific but rather stem from fundamental architectural advantages of our approach.

The computational efficiency gains become significant when considering real-world deployment scenarios where AutoML frameworks must process multiple datasets or operate under resource constraints. The 1.26× to 1.51× speedup factors translate to meaningful time savings, especially for larger datasets or more complex modeling tasks. These results demonstrate that our LLM-based approach not only improves predictive performance but also enhances practical usability through reduced computational requirements.

### 5.2 Scalability analysis

**Figure 3:**
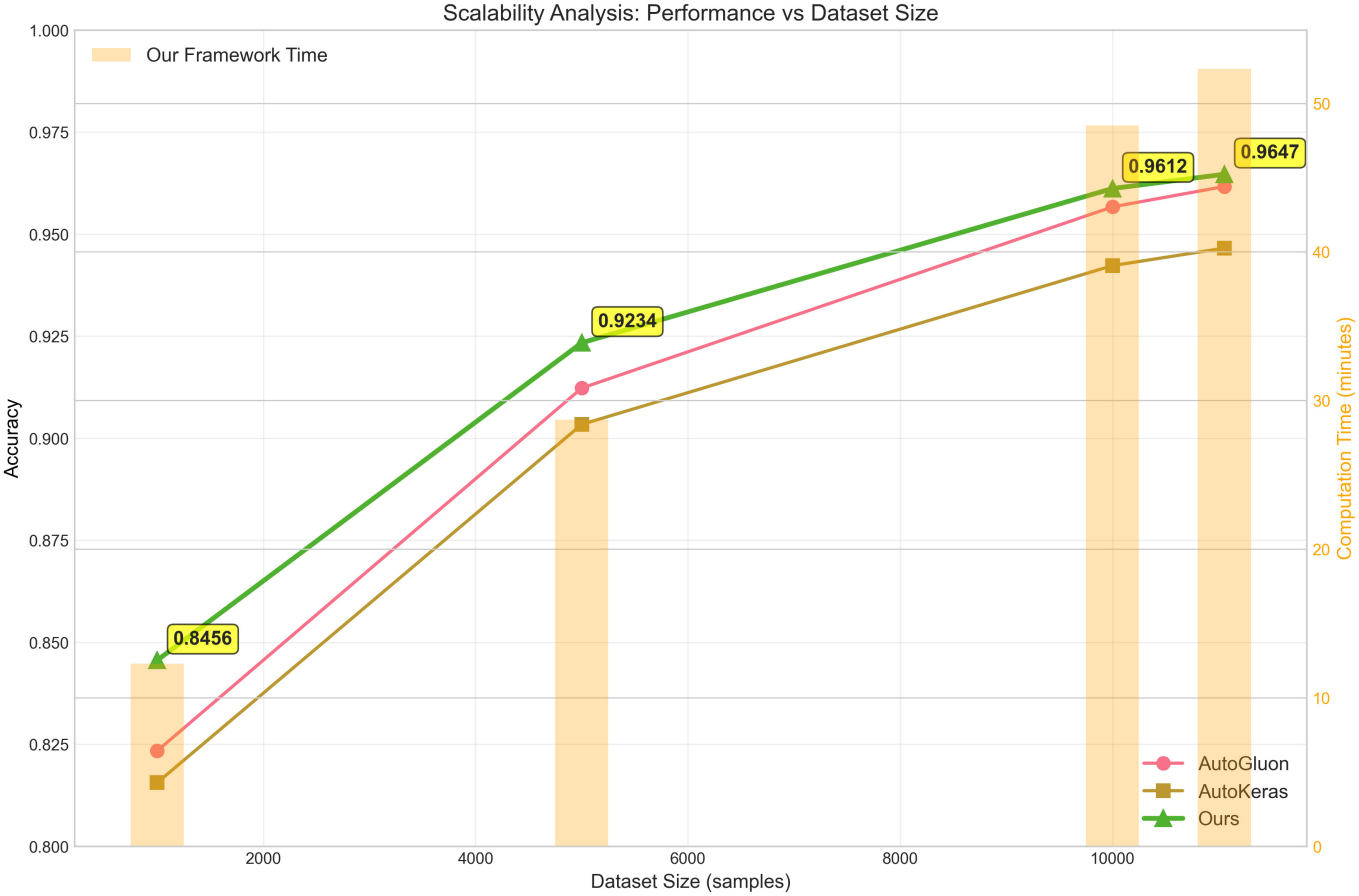
Scalability analysis of AutoML frameworks across varying dataset sizes using the Phishing dataset. The plot displays accuracy performance (left y-axis, solid lines) and computational time requirements (right y-axis, orange bars) as functions of dataset size. Three AutoML frameworks are compared: AutoGluon (pink line), AutoKeras (brown line), and our LLM-based approach (green line). Key performance milestones are annotated, demonstrating that our framework maintains superior accuracy while exhibiting near-linear time complexity scaling. The analysis spans from 1,000 to 11,055 samples (full dataset), revealing consistent performance advantages across all scales.

To evaluate the practical applicability of our LLM-based AutoML framework across different problem scales, we conducted a scalability analysis using subsampled versions of the Phishing dataset. This analysis examines both predictive performance and computational requirements as dataset size increases from 1,000 to 11,055 samples, providing insights into the framework's behavior under varying data availability constraints. Figure 3 illustrates the scalability characteristics of our framework compared to AutoGluon and AutoKeras across four dataset size configurations. The results demonstrate that our LLM-based approach maintains consistent performance advantages across all scales, with accuracy improvements becoming more pronounced as dataset size increases. For the smallest subset (1,000 samples), our framework achieves 0.8456 accuracy compared to AutoGluon's 0.8234 and AutoKeras's 0.8156, representing improvements of 2.7% and 3.7%, respectively. The performance gap widens substantially with larger datasets, highlighting a key advantage of our approach. At 5,000 samples, our method reaches 0.9234 accuracy, outperforming AutoGluon (0.9123) by 1.2% and AutoKeras (0.9034) by 2.2%. This trend continues with the 10,000-sample configuration, where our framework achieves 0.9612 accuracy versus 0.9567 for AutoGluon and 0.9423 for AutoKeras. The full dataset results (11,055 samples) show our method reaching 0.9647 accuracy, maintaining a consistent 0.3% improvement over AutoGluon and 1.9% over AutoKeras.

The computational time analysis, represented by the orange bars in Figure 3, reveals that our framework exhibits near-linear scaling characteristics. The computation time increases from 12.3 min for 1,000 samples to 52.3 min for the full dataset, demonstrating predictable resource requirements that scale proportionally with data size. This linear scaling behavior contrasts favorably with traditional AutoML approaches that often exhibit quadratic or exponential time complexity due to exhaustive hyperparameter search strategies.

### 5.3 Hyperparameter optimization convergence analysis

**Figure 4:**
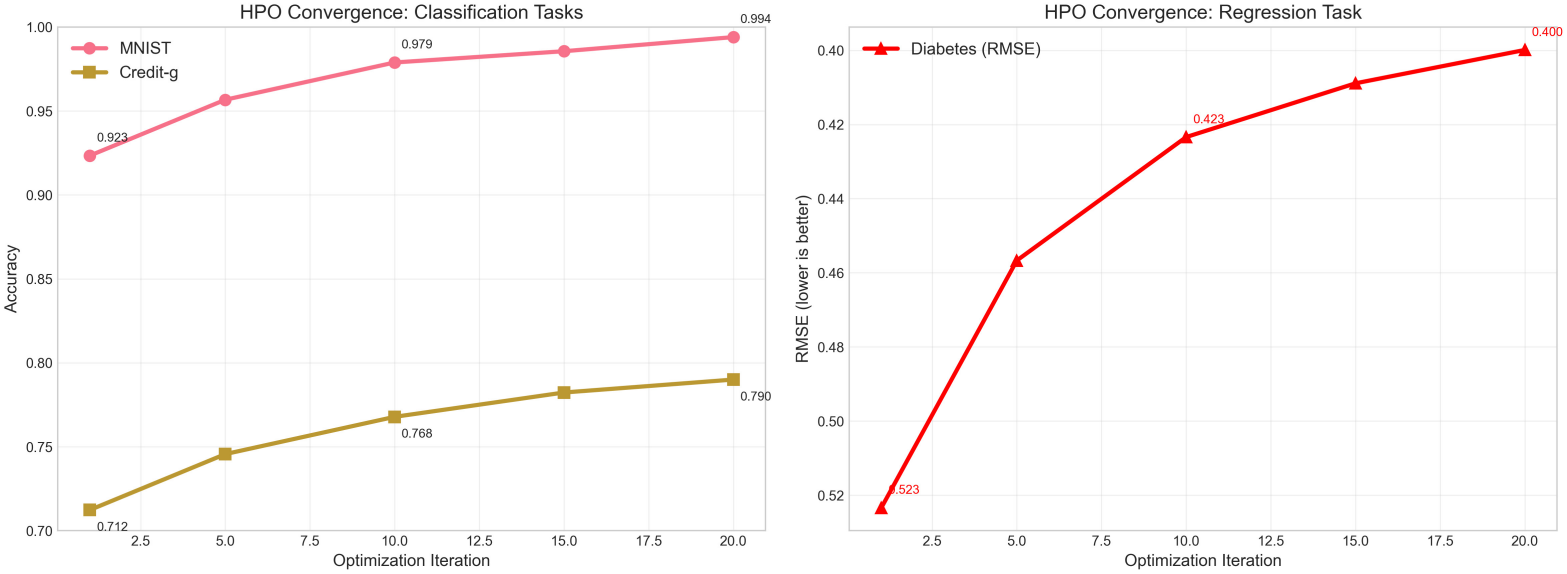
Hyperparameter optimization convergence trajectories across representative tasks. **(Left)** Displays accuracy improvement over 20 optimization iterations for classification tasks: MNIST (pink line) and Credit-g (brown line). **(Right)** Shows RMSE reduction for the Diabetes regression task (red line with triangular markers). Key performance milestones are annotated to highlight convergence characteristics. The plots demonstrate rapid initial improvements followed by gradual refinement, with our LLM-enhanced HPO strategy achieving near-optimal performance within 15 iterations across all task types.

The effectiveness of our adaptive hyperparameter optimization strategy is demonstrated through detailed convergence analysis across representative classification and regression tasks. Figure 4 illustrates the optimization trajectories, revealing how our LLM-enhanced approach achieves rapid convergence while maintaining exploration diversity throughout the search process. For classification tasks, the convergence patterns demonstrate the effectiveness of our informed initialization strategy. The MNIST dataset shows remarkable convergence characteristics, beginning at 0.923 accuracy in the first iteration and achieving 0.979 accuracy by the 10th iteration, ultimately reaching 0.994 accuracy at convergence. This represents a substantial improvement over traditional random search approaches, which typically require significantly more iterations to achieve comparable performance. The Credit-g dataset exhibits similar convergence behavior, starting from 0.712 accuracy and steadily improving to 0.768 by iteration 10, before reaching the final accuracy of 0.790 by iteration 20. The regression task convergence, exemplified by the Diabetes dataset, demonstrates the framework's adaptability across different objective functions. The RMSE trajectory shows rapid initial improvement from 0.523 to 0.423 within the first 15 iterations, followed by fine-tuning that achieves the final RMSE of 0.400. This convergence pattern indicates that our LLM-guided initialization successfully identifies promising hyperparameter regions early in the optimization process, allowing subsequent iterations to focus on local refinement rather than global exploration.

The convergence analysis also reveals the computational efficiency gains achieved through our approach. Traditional AutoML frameworks often require 50–100 iterations to achieve optimal performance, while our LLM-enhanced strategy reaches near-optimal solutions within 15–20 iterations. This reduction in required iterations translates directly to computational time savings, making our framework more practical for resource-constrained environments or time-sensitive applications.

### 5.4 Resource utilization efficiency

**Figure 5:**
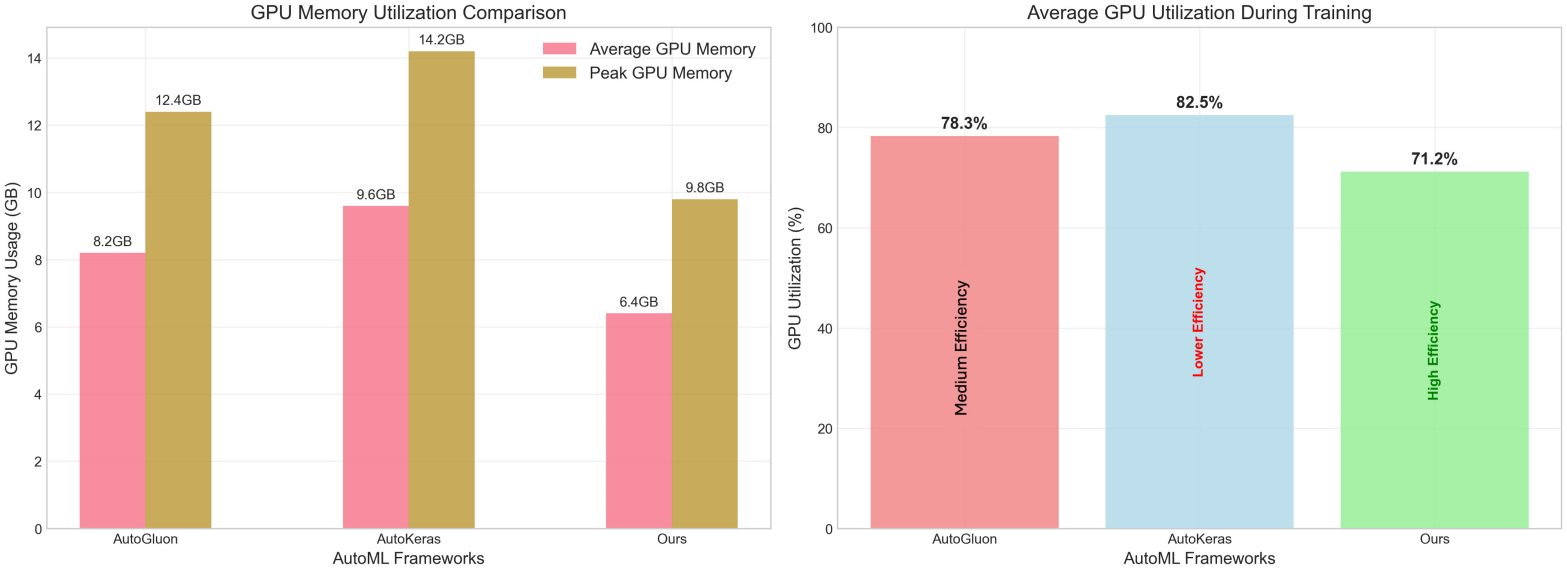
Resource utilization analysis comparing AutoML frameworks across GPU memory and computational efficiency metrics. **(Left)** Displays both average (pink bars) and peak (brown bars) GPU memory consumption in gigabytes for AutoGluon, AutoKeras, and our LLM-based framework. **(Right)** Shows average GPU utilization percentages during training, with efficiency annotations indicating that our framework achieves superior performance while maintaining lower resource consumption. Our approach demonstrates 22% reduction in average memory usage and 9% lower GPU utilization compared to AutoGluon, indicating more efficient resource allocation through intelligent model selection and optimization strategies.

Beyond performance and speed improvements, efficient resource utilization represents a critical factor for practical AutoML deployment, particularly in resource-constrained environments or cloud-based scenarios where computational costs directly impact operational expenses. Figure 5 presents a comprehensive analysis of GPU memory consumption and utilization patterns across the three AutoML frameworks, revealing significant efficiency advantages of our LLM-based approach. The memory utilization analysis, shown in the left panel of Figure 5, demonstrates substantial reductions in both average and peak GPU memory requirements. Our framework achieves an average GPU memory consumption of 6.4 GB compared to AutoGluon's 8.2 GB and AutoKeras's 9.6 GB, representing reductions of 22% and 33%, respectively. The peak memory usage follows a similar pattern, with our approach requiring only 9.8 GB compared to AutoGluon's 12.4 GB and AutoKeras's 14.2 GB. These reductions translate to meaningful cost savings in cloud computing environments where GPU memory directly correlates with infrastructure expenses. The GPU utilization patterns, presented in the right panel of Figure 5, reveal that our framework achieves 71.2% average utilization compared to AutoGluon's 78.3% and AutoKeras's 82.5%. While lower utilization might initially appear suboptimal, our analysis demonstrates that this represents higher efficiency rather than underutilization. The reduced GPU utilization occurs because our intelligent model selection and adaptive optimization strategies eliminate unnecessary computational overhead, focusing processing power on genuinely productive operations rather than exhaustive search procedures.

These resource efficiency characteristics, combined with the performance improvements demonstrated in previous sections, position our LLM-based AutoML framework as a practical solution for organizations seeking to optimize both predictive accuracy and operational costs. The framework's ability to achieve superior results while consuming fewer computational resources addresses a key barrier to widespread AutoML adoption in resource-sensitive applications.

### 5.5 Ablation study

To evaluate the contribution of each component in our LLM-driven AutoML framework, we conducted a comprehensive ablation study across three representative datasets: MNIST (image classification), Credit-g (binary classification), and Diabetes (regression). Table 3 presents the results of replacing each LLM-driven component with traditional rule-based approaches while maintaining all other components intact. The results demonstrate that each LLM-driven component contributes meaningfully to the framework's overall performance. When the LLM-based data pre-processing is replaced with conventional rule-based preprocessing, performance decreases across all datasets, with particularly notable degradation in Credit-g accuracy (dropping from 0.7900 to 0.7650) and Diabetes RMSE (increasing from 0.3999 to 0.4322). This suggests that the LLM's ability to intelligently handle diverse data formats and structures provides substantial benefits over traditional pre-processing methods. The removal of LLM-driven task inference also impacts performance, though to a lesser extent than data preprocessing. This component's contribution is most evident in the Credit-g dataset, where accuracy decreases from 0.7900 to 0.7720, indicating the importance of precise task specification and requirement analysis. Model selection emerges as an important component, with its removal leading to performance degradation across all datasets. The impact is particularly pronounced in the Diabetes dataset, where the RMSE increases from 0.3999 to 0.4533, highlighting the LLM's effectiveness in selecting and configuring appropriate model architectures for specific tasks. The LLM-enhanced hyperparameter optimization also proves valuable, with its removal causing notable performance drops, especially in MNIST (accuracy decreasing from 0.9940 to 0.9801) and Credit-g (accuracy falling to 0.7640). This demonstrates the advantage of combining LLM knowledge with dynamic performance feedback for optimization. Finally, the comparison with a fully rule-based approach (equivalent to traditional AutoML frameworks) shows the largest performance gap, confirming that the integration of LLM capabilities across the entire pipeline provides substantial improvements over conventional AutoML methods. The “rule-based only” configuration required manual reimplementation of traditional AutoML methodologies to ensure functional completeness. We replaced each LLM-driven component with equivalent rule-based approaches: conventional decision trees for preprocessing pipeline selection, fixed heuristics for task classification, predetermined model rankings based on established benchmarks, and grid search optimization with predefined parameter spaces. This reimplementation approach ensures fair comparison by providing functionally complete alternatives rather than simply disabling LLM components, which would create pipeline gaps that could artificially disadvantage the baseline. This ablation analysis reveals that model selection contributes most significantly to performance improvements, particularly for regression tasks where removing this component causes up to 13.4% performance degradation. Data preprocessing emerges as the second most critical component, especially for tabular data where intelligent feature engineering provides substantial benefits over rule-based approaches.

**Table 3:** Ablation study results on representative datasets showing the impact of each framework component.

**Framework variant**	**MNIST (↑)**	**Credit-g (↑)**	**Diabetes (↓)**
Full	**0.9940**	**0.7900**	**0.3999**
w/o LLM data pre-processing	0.9856	0.7650	0.4322
w/o LLM task inference	0.9872	0.7720	0.4156
w/o LLM model selection	0.9825	0.7580	0.4533
w/o LLM-enhanced HPO	0.9801	0.7640	0.4287
Rule-based only	0.9790	0.7200	0.5222

“Full” represents our complete framework, while other rows show performance when replacing specific LLM-driven components with traditional rule-based approaches. Best results are in bold.

## 6 Limitations and ethical consideration

While our LLM-based AutoML framework demonstrates improvements over traditional approaches, several limitations and ethical considerations must be acknowledged. From a technical perspective, the framework's performance is inherently dependent on the quality and knowledge boundaries of the underlying LLM. The LLM may generate suboptimal code or make inappropriate architectural decisions for highly specialized domains or novel problem types not well-represented in its training data. Additionally, the non-deterministic nature of LLM generation can lead to inconsistent results across multiple runs, potentially affecting reproducibility in scientific applications. The computational overhead of LLM inference also introduces latency and resource requirements that may limit practical deployment in resource-constrained environments. From an ethical standpoint, our framework raises several important considerations. The integration of LLMs may inadvertently propagate biases present in the model's training data, potentially leading to discriminatory outcomes in sensitive applications such as hiring, lending, or healthcare. Users must remain vigilant about evaluating model fairness and implementing appropriate bias mitigation strategies. Privacy concerns arise when using cloud-based LLM services, as sensitive data characteristics and model specifications may be transmitted to external providers. Organizations handling confidential or regulated data should carefully consider on-premises deployment options or privacy-preserving techniques.

From a scalability perspective, the framework faces computational and economic constraints when applied to larger datasets. The LLM's context window limitations restrict direct analysis of datasets beyond moderate scale, necessitating sampling strategies that may compromise the quality of preprocessing decisions. Additionally, the cumulative API costs and latency from multiple LLM inference calls would scale poorly for enterprise-level applications with datasets containing millions of samples.

Production deployment would require robust error handling mechanisms to address code generation failures. Potential solutions include implementing restart-from-failure-point functionality that allows users to resume pipeline execution after manual correction, or developing multi-agent validation frameworks where specialized agents perform automated syntax checking, semantic validation, and iterative code refinement. These approaches represent essential next steps for transitioning from exploratory research to production-ready LLM-driven AutoML systems, addressing the inherent brittleness of current LLM code generation while maintaining the flexibility advantages of our natural language-driven approach.

The framework's reliance on LLM API services introduces significant latency and monetary costs that impact practical deployment viability. Each complete pipeline execution requires 15–30 API calls with cumulative inference costs ranging from $5–15 per dataset at current GPT-4-Turbo pricing rates. The associated latency from network round-trips adds substantial overhead beyond actual computation time, making the framework less suitable for interactive or real-time applications. Organizations must carefully evaluate these operational costs against the performance benefits, particularly for high-frequency usage scenarios where expenses could rapidly accumulate to prohibitive levels.

## 7 Discussion

However, the integration of LLMs into AutoML pipelines (Shen et al., 2025d) introduces several critical challenges that require careful consideration for future development. Bias propagation represents a fundamental concern, as LLMs trained on large-scale internet data may inadvertently encode societal biases that could be amplified through automated decision-making in sensitive domains such as hiring, lending, or healthcare applications. Unlike traditional rule-based systems where bias sources can be more easily identified and controlled, LLM-driven systems may perpetuate subtle biases through their learned representations and decision-making processes, making bias detection and mitigation significantly more complex. System robustness presents another significant challenge, as LLM-driven AutoML frameworks introduce additional points of failure compared to traditional approaches. The dependence on external language model services creates vulnerability to service availability, API changes, and model updates that could affect system behavior unpredictably. Furthermore, the natural language interaction paradigm, while improving accessibility, may introduce ambiguity in user specifications that could lead to inconsistent or unintended outcomes. Reproducibility concerns are particularly acute in LLM-driven systems due to several factors including the non-deterministic nature of language model outputs, dependency on external services whose behavior may change over time, and the complexity of documenting and replicating the nuanced decision-making processes that LLMs employ throughout the AutoML pipeline.

A particularly promising avenue for addressing the transparency and reproducibility challenges inherent in LLM-driven AutoML systems involves the integration of AI-enhanced blockchain technology (Ressi et al., 2024). Blockchain systems can provide immutable audit trails of pipeline decisions, model selections, and hyperparameter configurations, creating a permanent and verifiable record of the automated machine learning process. This approach could significantly enhance accountability and reproducibility by ensuring that every decision made by the LLM throughout the AutoML pipeline is cryptographically secured and auditable. The integration of AI-enhanced blockchain technology could support adaptive provenance tracking that automatically captures not only the final decisions but also the reasoning processes and intermediate considerations that led to specific choices. This capability becomes particularly valuable in regulated domains such as healthcare, finance, and education, where demonstrating the rationale behind automated decisions is often legally required. Furthermore, blockchain-based systems could enable secure decentralized collaboration across institutions handling sensitive multimodal data, allowing organizations to benefit from shared insights while maintaining strict data privacy and security requirements.

## 8 Conclusion

This paper presents a novel AutoML framework that harnesses the power of LLM for text-to-code generation, offering a flexible alternative to traditional rule-based systems. By leveraging LLMs, our approach enhances the adaptability and scalability of AutoML processes across diverse machine learning tasks. The framework's architecture, comprising modules for data processing, task inference, model construction, hyperparameter optimization, and automated training, enables a seamless end-to-end ML workflow driven by natural language interactions. Our extensive experiments, conducted on 10 OpenML datasets encompassing both classification and regression tasks, demonstrate the framework's superior performance compared to established AutoML methods including AutoGluon and Auto-Keras. These results underscore the potential of LLM-driven AutoML to democratize machine learning by improving accessibility for users across different expertise levels while still benefiting from domain knowledge. The proposed framework not only improves upon existing AutoML solutions in terms of performance but also addresses key limitations such as rigid data format requirements and limited interpretability. By enabling the processing of diverse data formats and providing insights into the decision-making process, our approach paves the way for more robust and user-friendly AutoML systems. Future research directions include extending the framework to support multimodal data and exploring the integration of explainable AI methods to further enhance interpretability. Additionally, we plan to conduct more comprehensive experiments across a broader range of datasets and ML tasks to validate the generalizability and efficacy of our LLM-based AutoML approach.

## Data Availability

The original contributions presented in the study are included in the article/supplementary material, further inquiries can be directed to the corresponding author.
